# Aspects of Chemical Entropy Generation in Flow of Casson Nanofluid between Radiative Stretching Disks

**DOI:** 10.3390/e22050495

**Published:** 2020-04-25

**Authors:** Nargis Khan, Iram Riaz, Muhammad Sadiq Hashmi, Saed A. Musmar, Sami Ullah Khan, Zahra Abdelmalek, Iskander Tlili

**Affiliations:** 1 Department of Mathematics, The Islamia University of Bahawalpur, Bahawalpur 63100, Pakistan; nargiskhan49@gmail.com (N.K.); irramriaz1@gmail.com (I.R.); 2 Department of Mathematics, The Government Sadiq College Women University, Bahawalpur 63100, Pakistan; ms.hashmi@yahoo.com; 3 Industrial Engineering Department, The University of Jordan, Amman 11942, Jordan; saed_n_2000@yahoo.com; 4 Department of Mathematics, COMSATS University Islamabad, Sahiwal 57000, Pakistan; sk_iiu@yahoo.com; 5 Institute of Research and Development, Duy Tan University, Da Nang 550000, Vietnam; zahraabdelmalek@duytan.edu.vn; 6 Faculty of Medicine, Duy Tan University, Da Nang 550000, Vietnam; 7 Department for Management of Science and Technology Development, Ton Duc Thang University, Ho Chi Minh City 758307, Vietnam; 8 Faculty of Applied Sciences, Ton Duc Thang University, Ho Chi Minh City 758307, Vietnam

**Keywords:** entropy generation, stretching disk, thermal radiation, chemical reaction, shooting technique

## Abstract

The appropriate utilization of entropy generation may provoke dipping losses in the available energy of nanofluid flow. The effects of chemical entropy generation in axisymmetric flow of Casson nanofluid between radiative stretching disks in the presence of thermal radiation, chemical reaction, and heat absorption/generation features have been mathematically modeled and simulated via interaction of slip boundary conditions. Shooting method has been employed to numerically solve dimensionless form of the governing equations, including expressions referring to entropy generation. The impacts of the physical parameters on fluid velocity components, temperature and concentration profiles, and entropy generation number are presented. Simulation results revealed that axial component of velocity decreases with variation of Casson fluid parameter. A declining variation in Bejan number was noticed with increment of Casson fluid constant. Moreover, a progressive variation in Bejan number resulted due to the impact of Prandtl number and stretching ratio constant.

## 1. Introduction

Engineering systems’ efficiency decreases in the presence of irreversibilities. Heat transfer and fluid flow are irreversible processes and their irreversibility may be articulated in terms of entropy generation. Since rotating disks associated with non-Newtonian fluid flow and heat transfer have many important applications, such as in liquid-metal pumping, rotor-stator systems, oil recovery, hydraulic presses, centrifugal machinery, various electronic disks, shrouded-disk rotation, rotating motors, boilers, and plastic films and artificial fibers, they have received increasing attention over the last decade.

Gorder et al. [1] studied viscous fluid flow between stretching disks. Soid et al. [2] numerically analyzed the heat transfer characteristics induced by shrinking and stretching disks under the influence of magnetic field. Yin et al. [3] focused on the flow of nanofluid in rotating disks which radially stretched with uniform stretching rate. Another numerical investigation regarding nanofluid fluid configured by rotating disks was conducted by Sheikholeslami et al. [4]. Hashmi et al. [5] developed a mathematical model for Oldroyd-B confined by isothermal stretching disks, additionally featuring mixed convection and chemical reaction consequences. The radiative thermal analysis of Oldroyd-B fluid induced by two stretchable disks was securitized by Khan et al. [6]. Khan et al. [7] performed analytical computations for Maxwell fluid flow between stretching disks in the presence of a chemical reaction. 

Heating and/or cooling processes are encountered in almost every industrial system. Traditional heat transfer fluids have low thermal conductivity that seize their performance and put constraints on not only system compactness but also operational limits. The phenomenal thermal conductivity of nanofluids inherited from high thermal conductivity of tiny sized metallic particles suspended in the base fluid (such as water, oil, ethanol, and glycol), referred to as nanoparticles, drives their aggressive spread in industrial applications. The fundamental work on nanofluid was reported experimentally by Choi [8]. To analyze the slip mechanism of nanofluid, Buongiorno [9] introduced a mathematical model involving thermophoresis and Brownian motion effects. Ghadikolaei et al. [10] examined the Joule heating and nonlinear thermal radiation prospective in Casson nanofluid induced by stretched geometry. Khan and Shehzad [11] determined the thermophoretic aspects of nanofluid in third grade nano-material using a convergent technique. Alwatban et al. [12] interpreted the role of Wu’s slip in Eyring Powell nanofluid with additional impact of activation energy. A flow model regarding bioconvection of Oldroyd-B nanoliquid in existence of activation energy generated by stretched cylinder was suggested by Tlili and co-workers [13]. Waqas et al. [14] reported some biofuels application associated with the flow of nanoparticles in presence of gyrotactic microorganisms. They used a second grade viscoelastic nanofluid model where the numerical solution had been calculated via built-in bvp4c algorithm. A nanofluid study of third grade fluid flow with the impact of thermal radiation, viscous dissipation, and slip consequences was explored by Abdelmalek et al. [15]. Eid et al. [16] carried out electromagnetic features in blood flow carbon nanotubes in a porous circular cylinder. The rheological features of Cassonnanofluid in presence of activation energy has been analyzed by Shah et al. [17]. Eid [18] investigated the flow of Siskonanofluid induced by a convectively heated surface. The thermal aspects of MnFe_2_O_4_nanoparticles immersed in non-Newtonian fluid were suggested by Shaw et al. [19]. Sheremet et al. [20] utilized the significance of nanoparticles in cavity where corner and top walls are assumed to be heated. In another useful contribution, Sheremet and Pop [21] implemented the famous LTNE and Buongiorno’s models while examining the local heater size and position effects configured a porous cavity. The entropy generation and thermal aspects of TiO_2_ nanoparticles for flow of micropolar in a porous medium were examined numerically by Zaib et al. [22]. Sheikholeslami et al. [23] studied the significance of nanoparticles for convective flow in porous chambers additionally impacted with thermal radiation and magnetic force. Another investigation based on utilization of nano-materials in a baffled U-shaped enclosure was numerically simulated by Ma et al. [24]. The impact of Lorentz force in porous annulus in presence of CuO-H_2_O nanofluid was investigated by Sheikholeslamiet al. [25]. Bondarenko et al. [26] reported thermal features of Al_2_O_3_/H_2_O nano-material in a cavity with a feature of heat-generating element. Selimefendigil et al. [27] analyzed the pulsating flow of ferrofluid with appliance of mixed convection features. Some further investigation deals with applications of nano-materials, which can be seen in references [28,29,30,31].

The thermodynamic optimization of various thermal engineering processes has improvement of the sustainability and efficiency of emerging technologies in recent decades. Various thermal extrusion systems and heat transportation mechanisms are designed based on the laws of thermodynamics. According to the first law of thermodynamics, energy can be transformed within different systems or mediums instead of being lost. However, this law fails to justify the irreversibilities (entropy generation). On the other hand, the second thermodynamics law itemizes the collection and available energy consumption and reduces the energy loss, and subsequently improves the fundamental thermal efficiency of the heat transportation system. Abolbashari et al. [32] examined the entropy generation aspects in unsteady nanofluid flow coffered by a moving surface. Kumar et al. [33] evaluated the effects of entropy generation for the flow of viscoelastic nano-material under the influence of transient convective dissipation. Another numerical investigation for determination of entropy generation for nanofluid has been explored by Rana et al. [34]. Aghakhani et al. [35] worked on the thermal aspects for natural convective flow alumina/water nanoparticles in presence of entropy generation features. Seyyedi et al. [36] examined the magnetic field and entropy generation impact to examine the heat transfer analysis in L-shaped enclosures. Salimi et al. [37] incorporated the features of entropy generation and heat sink for 3D jet flow under the assumptions of local thermal non-equilibrium constraints. The flow of chemically reactive non-Newtonian liquid due to stretched surface in presence of entropy generation phenomenon was recently considered by Khan et al. [38]. Mustafa [39] investigated slip effects in nanofluid flow induced by a rotating disk. Arikoglu et al. [40] investigated the entropy generation features in slip flow of viscous fluid due to rotating disk.

Recently, special interest has developed towards the flow of nanoparticles because of their diverse industrial and commercial applications, like energy generation, improvement of the thermal extrusion phenomenon, development of manufacturing processes, etc. In addition to this, the consumption of available energy and reduced energy loss is another novel aspect useful in various engineering applications and other industries to improve the thermal efficiency of systems. The utilization of entropy generation enables the minimization of available energy loss of performance systems. Keeping such motivations in mind, the present investigation presents the effects of entropy generation in flow of Casson nanofluid induced by stretching disks.

After carefully examining the above cited work, we note that entropy generation features in thermally developed flow of Casson nanofluid induced by two porous stretching disks have not been reported yet. Therefore, the current analysis aims to fill this gap. Additionally, the interesting features of magnetic field, heat absorption/generation and chemical reaction are also incorporated. The present research is an extension of that of Arikoglu et al. [40]. It integrated the Casson nanofluid model. Also, the novel features of thermal radiation, heat source/sink, and chemical reaction are incorporated. The analysis has been performed over porous stretching disks in contrast to simple stretching disks, which addresses modern engineering applications in the fields of material engineering, biomedical separation devices, petroleum engineering, distillation towers, jet engines, and atmospheric flows. The governing equations for current flow situations are constituted and tackled by employing the famous numerical shooting technique. Different flow parameters are graphically impacted with relevant physical significant. 

## 2. Mathematical Analysis

**Figure 1:**
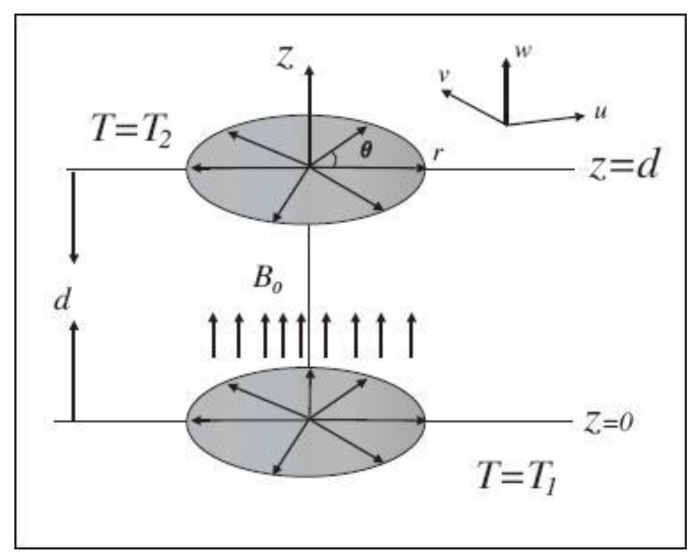
Schematic diagram and coordinate system.

In order to model the equations for flow analysis, we consider a steady two-dimensional axisymmetric flow of an incompressible, electrically conducting Casson nanofluid between two stretchable disks. The magnetic field effects with magnetic field strength $B_{0}$ are applied vertically to the surface shown in Figure 1. Here (u, v, w) and (r,  θ,  z) are assumed to be velocity components and cylindrical coordinates. The flow is described in cylindrical coordinates (r, θ,  z) where z is chosen as the perpendicular axis. The lower disk is static at the plane z=0. The governing equations for current flow problem are [1,5,6,7];

(1) $$\frac{\partial u}{\partial r}+\frac{u}{r}+\frac{\partial w}{\partial z}=0,$$

(2) $$u\frac{\partial u}{\partial r}+w\frac{\partial u}{\partial z}=-\frac{1}{\rho }\frac{\partial p}{\partial r}+\nu \left(\frac{1+\beta }{\beta }\right)\left(\frac{\partial^{2}u}{\partial r^{2}}+\frac{1}{r}\frac{\partial u}{\partial r}-\frac{u}{r^{2}}+\frac{\partial^{2}u}{\partial z^{2}}\right)-\frac{\sigma B_{0}{}^{2}}{\rho }u-\frac{\nu u}{k},$$

(3) $$u\frac{\partial w}{\partial r}+w\frac{\partial w}{\partial z}=-\frac{1}{\rho }\frac{\partial p}{\partial z}+\nu \left(\frac{1+\beta }{\beta }\right)\left(\frac{\partial^{2}w}{\partial r^{2}}+\frac{1}{r}\frac{\partial w}{\partial r}+\frac{\partial^{2}w}{\partial z^{2}}\right),$$

(4) $$u\frac{\partial T}{\partial r}+w\frac{\partial T}{\partial z}=\alpha \left(\frac{\partial^{2}T}{\partial r^{2}}+\frac{1}{r}\frac{\partial T}{\partial r}+\frac{\partial^{2}T}{\partial z^{2}}\right)+\tau \left[\begin{array}{c} D_{B}\left(\frac{\partial C}{\partial r}\frac{\partial T}{\partial r}+\frac{\partial C}{\partial z}\frac{\partial T}{\partial z}\right)\\ +\frac{D_{T}}{T_{m}}\left\{{\left(\frac{\partial T}{\partial r}\right)}^{2}+{\left(\frac{\partial T}{\partial r}\right)}^{2}\right\} \end{array}\right]-\frac{1}{\rho c_{p}}\frac{\partial q_{r}}{\partial z}+\frac{Q_{0}}{\rho c_{p}}\left(T-T_{1}\right),$$

(5) $$u\frac{\partial C}{\partial r}+w\frac{\partial C}{\partial z}=D_{B}\left(\frac{\partial^{2}C}{\partial r^{2}}+\frac{1}{r}\frac{\partial C}{\partial r}+\frac{\partial^{2}C}{\partial z^{2}}\right)+\frac{D_{T}}{T_{m}}\left(\frac{\partial^{2}T}{\partial r^{2}}+\frac{1}{r}\frac{\partial T}{\partial r}+\frac{\partial^{2}T}{\partial z^{2}}\right)-k_{0}\left(C-C_{1}\right),$$

For Equations (1)–(5), the slip boundary conditions are developed as:

$u=ar+b_{1}\frac{\partial u}{\partial z},\;\;\;\;\;\;\;\;w=0,\;\;\;\;\;\;\;\;P=\frac{a\mu Br^{2}}{4d^{2}},\;\;\;\;\;\;\;\;at\;\;z=0,$

$u=cr-b_{2}\frac{\partial u}{\partial z},\;\;\;\;\;\;\;\;w=0,\;\;\;\;\;\;\;\;p=0,\;\;\;\;\;\;\;\;\;\;\;\;\;\;\;\;at\;z=d,$

$T=T_{0},\;\;\;\;\;\;\;\;at\;\;\;\;z=0,\;\;\;\;\;\;\;\;T=T_{1},\;\;\;\;\;\;\;\;at\;z=d,$

(6) $$C=C_{1},at\;\;\;\;z=0,C=C_{2},at\;\;\;z=d.$$

where µ isthe dynamic viscosity, ρ denotes density, β is the parameter of Casson fluid, p is the pressure, σ is the electrical conductivity, $B_{0}$ is the magnetic field strength, k is the permeability of porous medium, T is the temperature, C is the concentration, α is the thermal diffusivity, $c_{p}$ reflects the specific heat at constant pressure, $Q_{0}$ is the heat generation parameter, $D_{T}$ is the thermophoretic diffusion coefficient, $D_{B}$ is the Brownian diffusion coefficient, τ is the ratio of heat capacity, $k_{0}$ is the reaction constant, $T_{m}$ is the mean temperature, while Nb is the Brownian motion coefficient. In view of Rossel and approximation, the radiative heat flux $\left(q_{r}\right)$ is written as:

(7) $$q_{r}=-\frac{4}{3}\frac{\sigma^{*}}{k^{*}}\frac{\partial T^{4}}{\partial z},$$

where $k^{*}$ is the coefficient of Rossel and mean absorption, $\sigma^{*}$ is the constant of Stefan–Boltzmann. By expanding $T^{4}$ about free stream temperature $T_{0}$ as follows:

(8) $$T^{4}=T_{0}{}^{3}+4T_{0}{}^{3}\left(T-T_{0}\right)+6\left(T-T_{0}{}^{2}\right)+\ldots ,$$

For further analysis, the temperature gradient within the flow is assumed to be small and subsequently the higher order terms can be ignored i.e.,

(9) $$T^{4}=4T_{0}{}^{3}\left(T-T_{0}\right),$$

By using Equation (9) in Equation (7), we have

(10) $$q_{r}=-\frac{16}{3}\frac{\sigma^{*}}{k^{*}}T^{3}\frac{\partial T}{\partial z}.$$

Now let us introduce similarity transforms as follows [5,6,7]:

$u=arH^{\prime }\left(\eta \right),w=adH\left(\eta \right),\eta =\frac{z}{d},$

(11) $$P=a\mu \left(P\left(\eta \right)+\frac{\beta r^{2}}{4d^{2}}\right),\theta \left(\eta \right)=\frac{T-T_{0}}{T_{1}-T_{0}},\emptyset \left(\eta \right)=\frac{C-C_{0}}{C_{1}-C_{0}}.$$

By using similarity transforms in Equations (1) to (5), the following transformed governing equations are obtained

(12) $$\left(\frac{1+\beta }{\beta }\right)H^{\prime \prime \prime }-Re({H^{\prime }}^{2}+HH^{\prime \prime })-Re\left(M+S\right)H^{\prime }=0,$$

(13) $$\left(1+\frac{4}{3}Rd\right)\theta^{\prime \prime }-RePrH\theta^{\prime }+Pr\lambda \theta +N_{b}Pr\theta^{\prime }\emptyset^{\prime }=0,$$

(14) $$\emptyset^{\prime \prime }-PrLeReH\emptyset^{\prime }+\frac{N_{t}}{N_{b}}\theta^{\prime \prime }-PrLeReK_{1}\emptyset =0,$$

(15) $$P^{\prime }=\left(\frac{1+\beta }{\beta }\right)H^{\prime \prime }-ReH^{\prime }H,$$

H(0)=0, H(1)=0, P(0)=0,

(16) $$H^{\prime }\left(0\right)=-2+\lambda_{1}H^{\prime \prime }\left(0\right),\;\;\;\;H^{\prime }\left(1\right)=-2-\lambda_{2}H^{\prime \prime }\left(1\right),$$

θ(0)=0,    θ(1)=1,∅(0)=0,            ∅(1)=1,

where the dimensionless parameters are defined as

$\lambda =\frac{Qd^{2}}{\nu },Rd=\frac{KK^{*}}{4\sigma^{*}}T^{3},Pr=\frac{\nu }{\alpha }\;,Le=\frac{\alpha }{D_{B}},M=\frac{\sigma B_{0}{}^{2}}{\rho a},Re=\frac{ad^{2}}{\nu },$

$Nb=\tau D_{B}\left(\frac{C_{2}-C_{1}}{\nu }\right),\;Nt=\tau \frac{D_{T}}{T_{m}}\left(\frac{T_{2}-T_{1}}{\nu }\right),\;\lambda_{1}=\frac{b_{1}}{d},\;\lambda_{2}=\frac{b_{2}}{d},\;S=\frac{\nu }{ka},\;K_{1}=\frac{k_{0}}{a}.$

In the above expression, γ is stretching ratio, λ is heat source parameter, Rd is thermal radiation parameter, Re is Reynolds number, Pr is Prandtl number, Le is the Lewis number, M is parameter of magnetic, Nb is parameter of Brownian motion, $D_{B}$ is Brownian diffusion parameter, Nt is thermophoretic parameter, $\lambda_{1}$ and $\lambda_{2}$ are slip lengths, S is porosity parameter, and $D_{T}$ is thermophoresis diffusion coefficient.

## 3. Entropy Generation Equation

The expressions for local entropy generation volumetric rate for Casson nanofluid are given by

(17) $$S_{G}=\frac{K}{T_{0}{}^{2}}{\left[\nabla T\right]}^{2}+\frac{\mu }{T_{0}}\left(\frac{1+\beta }{\beta }\right)\Phi +\frac{\mu }{c_{1}}{\left(\frac{\partial C}{\partial z}\right)}^{2}+\frac{\mu }{c_{1}}\left(\frac{\partial T}{\partial z}\right)\left(\frac{\partial C}{\partial z}\right)+\frac{\sigma B_{0}{}^{2}}{T_{0}}u^{2}+\frac{\mu }{KT_{0}}u^{2},$$

where μ and K are the viscosity and thermal conductivity, $T_{0}$ is the reference temperature, and Φ is the viscous dissipation. In Equation (17), the term Φ can be written as:

(18) $$\Phi =2\left[{\left(\frac{\partial u}{\partial r}\right)}^{2}+\frac{u^{2}}{r^{2}}+{\left(\frac{\partial w}{\partial z}\right)}^{2}\right]+\frac{1}{r^{2}}{\left(\frac{\partial w}{\partial \theta }\right)}^{2}+{\left(\frac{\partial u}{\partial z}\right)}^{2}+\frac{1}{r^{2}}{\left(\frac{\partial w}{\partial \theta }\right)}^{2}.$$

Using Equation  (18) in Equation  (17) yields

(19) $$S_{G}=\frac{K}{T_{0}{}^{2}}{\left(\frac{\partial T}{\partial Z}\right)}^{2}+\frac{\mu }{T_{0}}\left(\frac{1+\beta }{\beta }\right)\left\{\begin{array}{c} 2\left[{\left(\frac{\partial u}{\partial r}\right)}^{2}+\frac{u^{2}}{r^{2}}+{\left(\frac{\partial w}{\partial z}\right)}^{2}\right]+\\ \frac{1}{r^{2}}{\left(\frac{\partial w}{\partial \theta }\right)}^{2}+{\left(\frac{\partial u}{\partial z}\right)}^{2}+\frac{1}{r^{2}}{\left(\frac{\partial w}{\partial \theta }\right)}^{2} \end{array}\right\}\;+\frac{\mu }{C_{1}}{\left(\frac{\partial C}{\partial z}\right)}^{2}+\frac{\mu }{C_{1}}\left(\frac{\partial T}{\partial z}\right)\left(\frac{\partial C}{\partial z}\right)+\frac{\sigma B_{0}{}^{2}}{T_{0}}u^{2}+\frac{\mu }{KT_{0}}u^{2},$$

The characteristics entropy generation is defined as

(20) $$S_{G_{0}}=\frac{k\left(T_{1}-T_{0}\right)}{d^{2}T_{0}},$$

After inserting Equation  (11) into Equation  (17), the following equation for entropy generation number will be obtained

(21) $$N_{G}=\alpha {\theta^{\prime }}^{2}+Br\left\{\left(\frac{1+\beta }{\beta }\right)\left(\frac{3}{\delta^{2}}{H^{\prime }}^{2}+{H^{\prime \prime }}^{2}\right)-\frac{1}{4}Re\left(M+S\right){H^{\prime }}^{2}\right\}+\lambda_{3}\frac{\epsilon }{\alpha^{2}}{\emptyset^{\prime }}^{2}+\lambda_{4}\epsilon \theta \emptyset^{\prime },$$

where

$Br=PrEc,\;\;Ec=\frac{a^{2}r^{2}}{Cp\left(T_{1}-T_{0}\right)},\epsilon =\frac{C_{2}-C_{1}}{C_{1}}$, $\lambda_{3}=\frac{\mu c_{1}}{k},\lambda_{4}=\frac{\mu T_{0}}{k},\delta =\frac{r}{d}$.

In the above expression, Br is the Brinkman number and Ec is the Eckert number. The Bejan number Be is another alternative irreversibility distribution parameter. This is the ratio between entropy generations due to heat transfer to the total entropy generations. In a dimensionless form, Bejan number is given as follows:

(22) $$Be=\frac{Entropy\;generation\;due\;to\;heat\;transfer\;}{Total\;entropy\;generation},$$

(23) $$Be=\frac{\alpha {\theta^{\prime }}^{2}}{\alpha {\theta^{\prime }}^{2}+Br\left\{\left(\frac{1+\beta }{\beta }\right)\left(\frac{3}{\delta^{2}}{H^{\prime }}^{2}+{H^{\prime \prime }}^{2}\right)-\frac{1}{4}Re\left(M+S\right){H^{\prime }}^{2}\right\}}.$$

## 4. Results and Discussion

The system of Equations (12)–(14), along with boundary conditions, Equation (16), has been solved numerically by employing the shooting method. The results are validated by comparing present numerical values with those generated by Mustafa [39], and presented in Table 1. A convincible accuracy of results has been found between both studies. In this section, physical explanation of flow parameters, like the stretching ratio γ, Reynold number, Reynold number Re, Prandtl number Pr, magnetic parameter M, thermophoretic parameter Nt, thermal radiation parameter Rd, Lewis number Le, and Brownian motion parameter Nb, is presented graphically. Each parameter is varied while other parameters are kept constant, i.e., M=1, β=0.1, γ=0.3, S=0.5, Nt=0.5, λ=0.5, $Le=1.5,\;Nb=0.7,\;K_{1}=0.3$, and Pr=0.5. 

**Figure 2:**
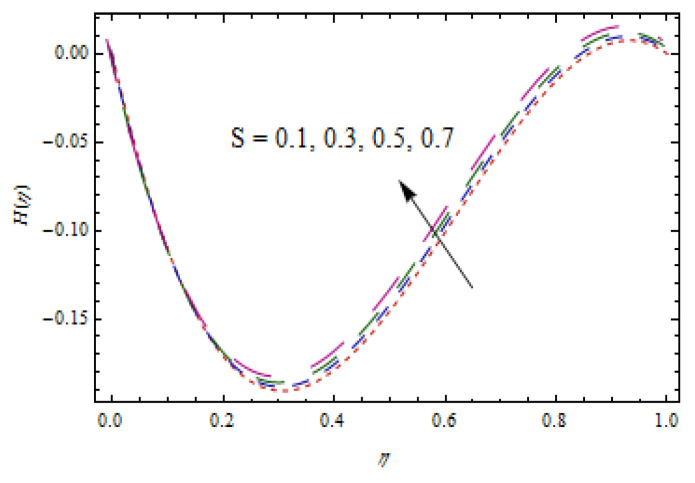
Impact of S on H(η).

**Figure 3:**
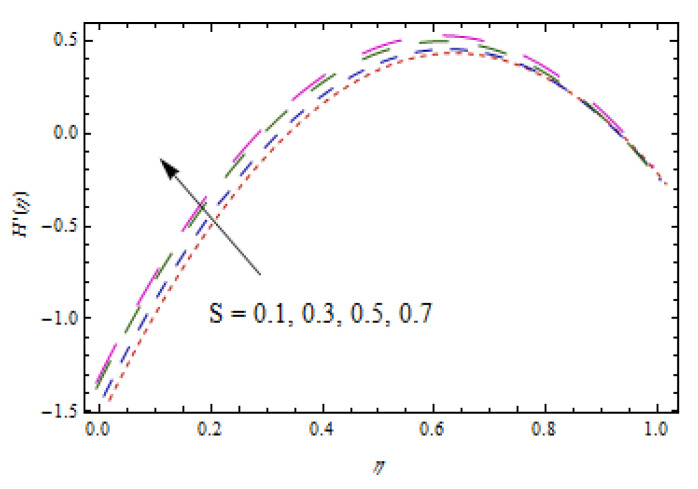
Impact of S on $H^{\prime }\left(\eta \right)$.

**Figure 4:**
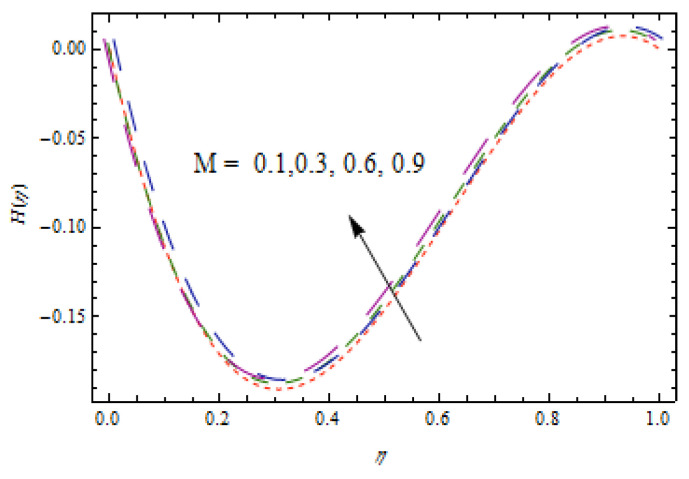
Impact of M on H(η).

**Figure 5:**
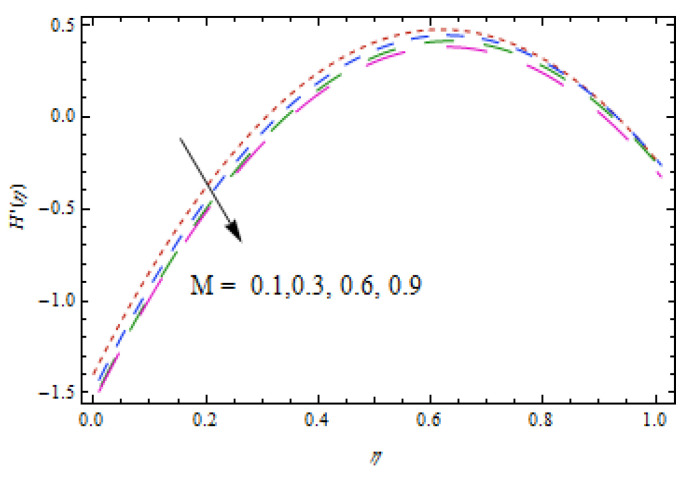
Impact of M on $H^{\prime }\left(\eta \right)$.

**Figure 6:**
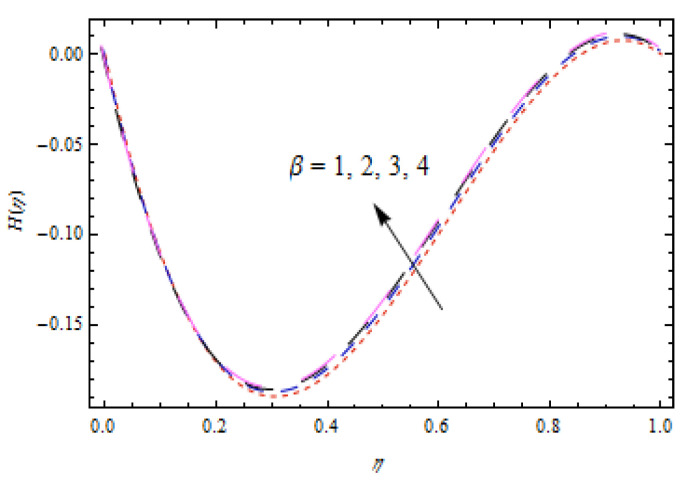
Impact of β on H(η).

**Figure 7:**
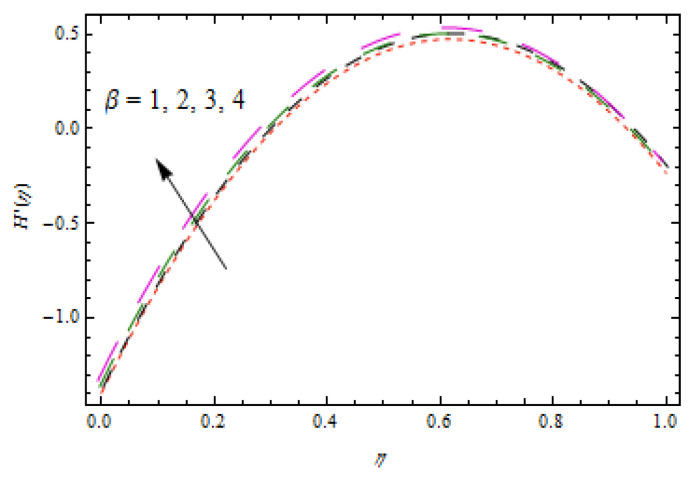
Impact of β on $H^{\prime }\left(\eta \right)$.

**Figure 8:**
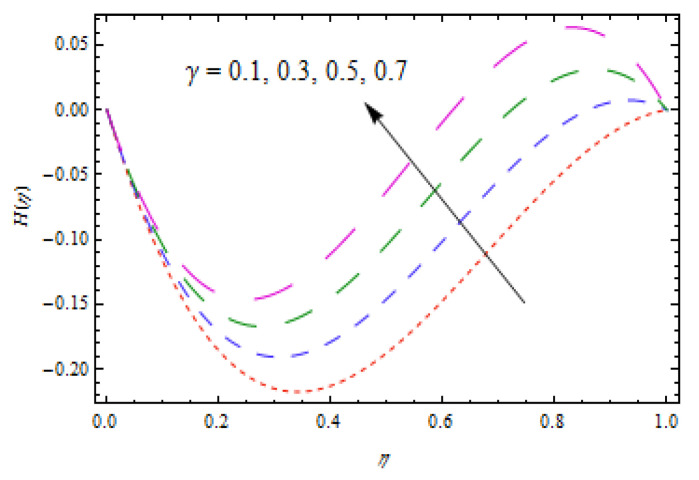
Impact of γ on H(η).

**Figure 9:**
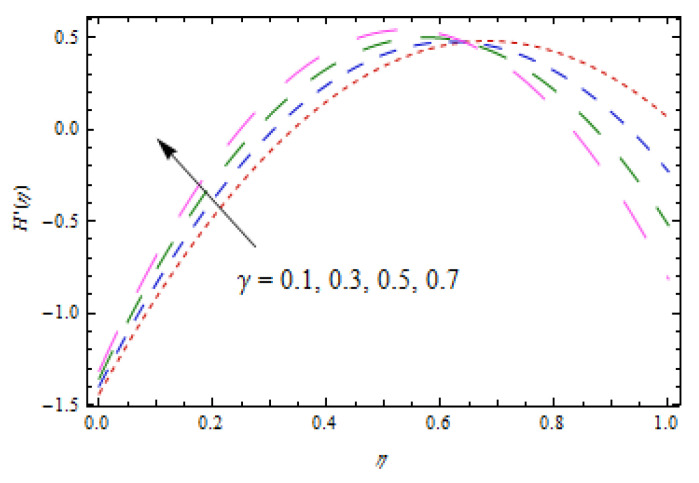
Impact of γ on $H^{\prime }\left(\eta \right)$.

Figure 2, Figure 3, Figure 4, Figure 5, Figure 6 and Figure 7 show the influence of porosity parameter, magnetic parameter, and Casson fluid constant on axial velocity component H(η) and radial velocity component $H^{\prime }\left(\eta \right)$. In Figure 2 and Figure 3, velocity profiles increase for rising values of porosity parameter S. The physical justification of such an increasing trend may be attributed to the involvement of the permeability of the porous medium. Figure 4 shows that the axial velocity component is a growing function of magnetic parameter M. It is seen that the thickness of thermal boundary layer surges with the implementation of magnetic field. Figure 5 shows that radial velocity component is decreasing function of magnetic parameter due to stretching disks. Physically, a change in magnetic number is associated with the Lorentz force, which is of resistive nature and subsequently decreases the nanoparticles’ velocity. Figure 6 and Figure 7 show that increment of Casson fluid parameter β leads to a progressive velocity distribution. The physical aspect of such trend is due to involvement of yield stress which is associated with Casson fluid parameter β. The graphical explanations presented in Figure 8 and Figure 9 reveal that the axial and radial velocity component increases by varying stretching ratio γ.

**Figure 10:**
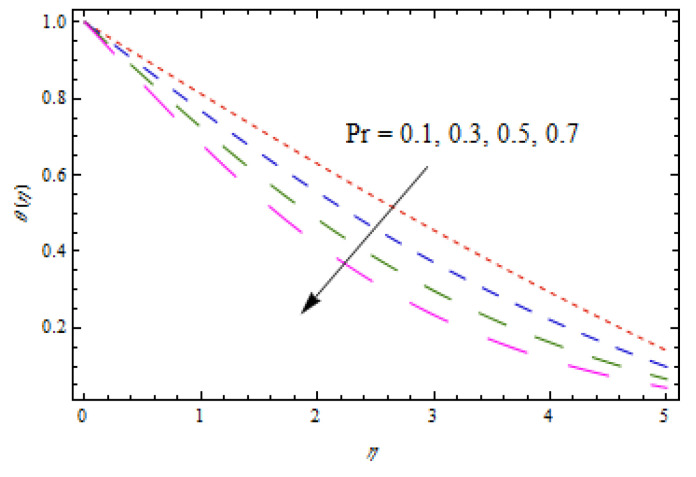
Impact of Pr on θ(η).

**Figure 11:**
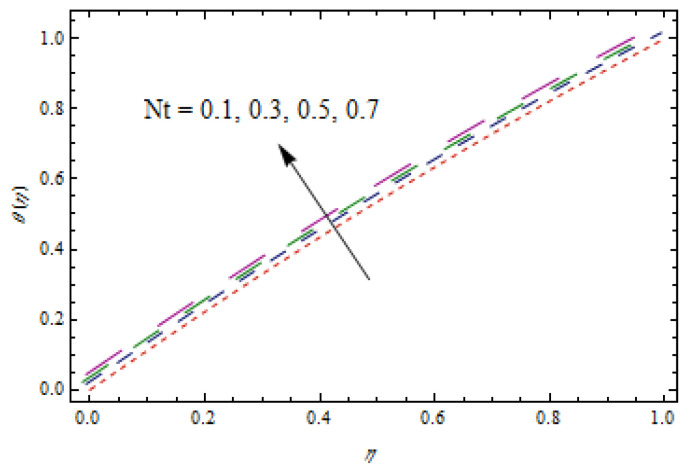
Impact of Nt on θ(η).

**Figure 12:**
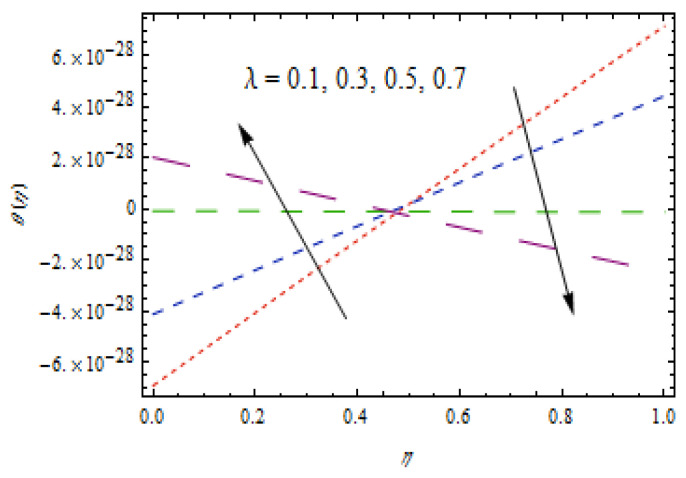
Variation in θ(η) for heat source case.

**Figure 13:**
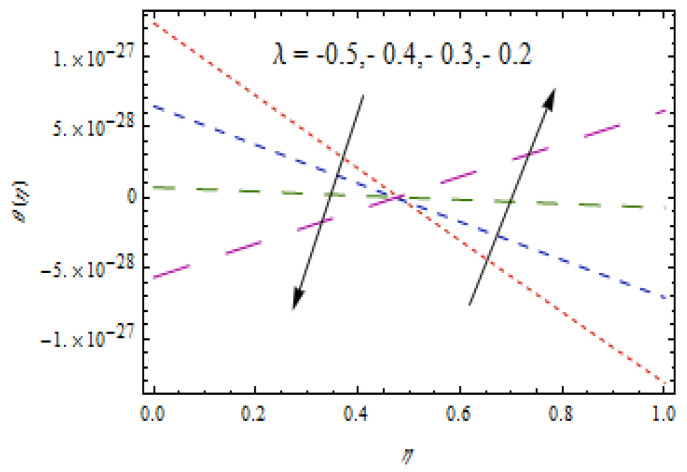
Variation in θ(η) for heat sink case.

Figure 10, Figure 11, Figure 12 and Figure 13 show the influence of Prandtl number Pr, thermophoretic parameter Nt, and heat source/sink parameter λ on temperature profiles θ(η). Figure 10 shows a declining temperature distribution θ for apparent values of Prandtl number Pr. Such a decline in temperature profile due to peak values of Prandtl number is due toa weaker thermal diffusivity. Therefore, proper values of Prandtl number play a frequent role in controlling the heating and cooling processes. Figure 11 depicts the consequence of thermophoretic parameter Nt on θ. The thermophoresis phenomenon has a significant contribution in many industries. The thermophoresis is a migration process of heated fluid particles towards the cold region, due to which the temperature increases. From Figure 12, an increasing temperature profile is resulted for heat source parameter (λ>0). However, for heat sink case (λ<0), opposite observations are obtained. Physically, an improved temperature due to heat source is associated with addition of heat to the system. However, for the heat sink case, heat is removed from the system which reduces the temperature.

**Figure 14:**
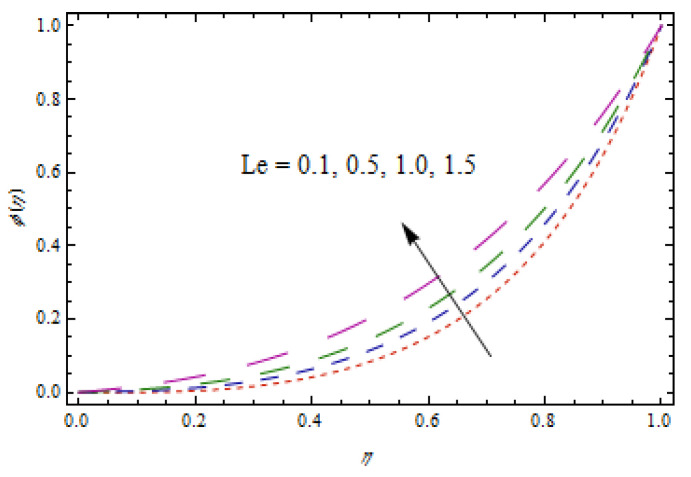
Impact of Le on ϕ(η).

**Figure 15:**
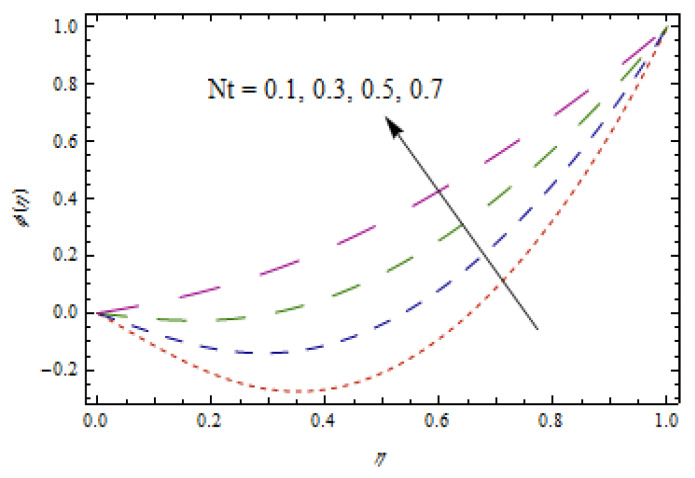
Impact of Nt on ϕ(η).

**Figure 16:**
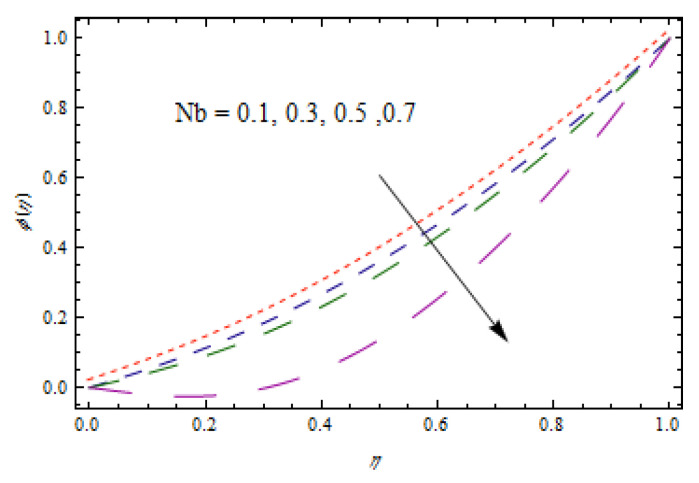
Impact of Nb on ϕ(η).

**Figure 17:**
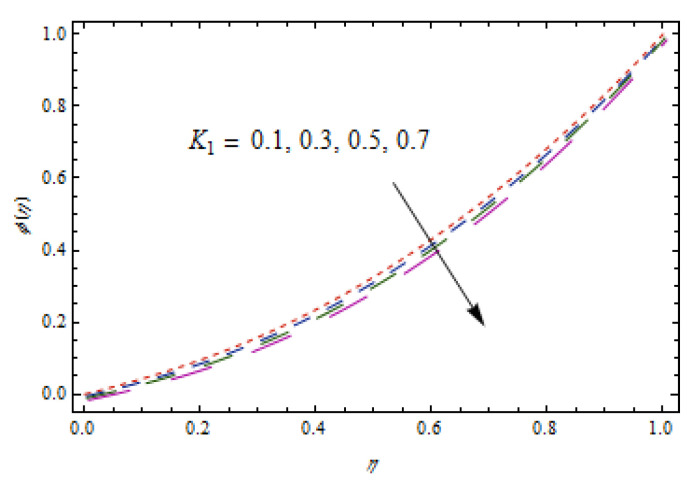
Impact of $K_{1}$ on ϕ(η).

**Figure 18:**
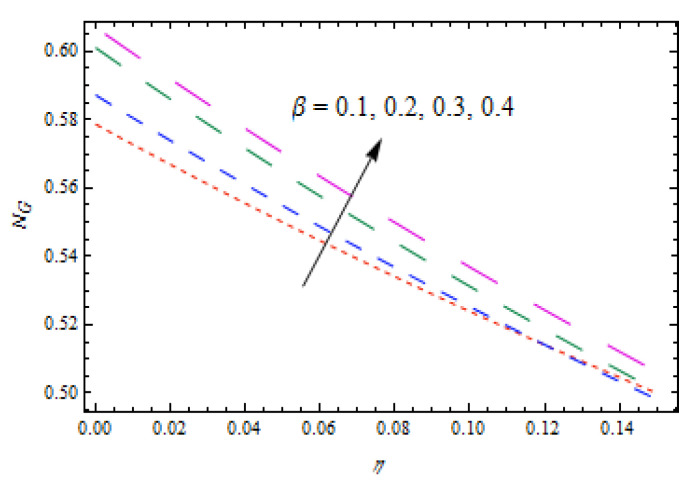
Impact of β on $N_{G}$.

**Figure 19:**
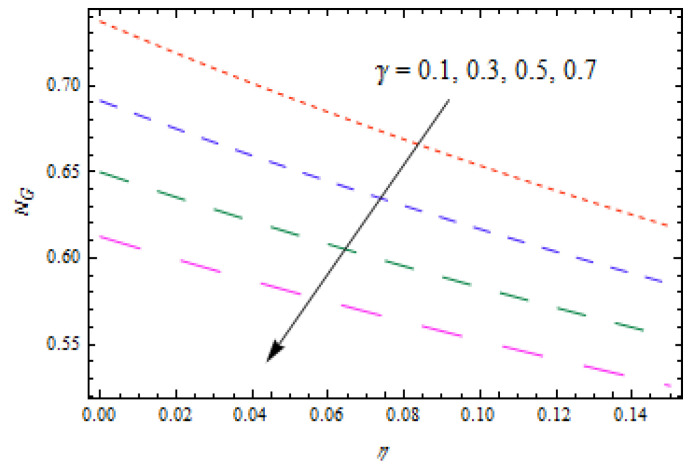
Impact of γ on $N_{G}$.

Figure 14, Figure 15, Figure 16 and Figure 17 predict the influence of the Lewis number Le, thermophoretic parameter Nt, Brownian motion parameter Nb, and reaction parameter $K_{1}$ on concentration profiles ϕ(η). Figure 14 and Figure 15 show that concentration profiles increase with the increase in Le and Nt. Physically, the Lewis number is associated with the mass diffusion coefficient. The higher variation in Lewis number corresponds to low mass diffusion which declines the concentration of nanoparticles. Figure 16 and Figure 17 show decreasing behavior of concentration profile by increasing the value of Brownian motion parameter Nb and chemical reaction parameter $K_{1}$.The Brownian constant Nb occupies a reverse relation with dimensionless concentration Equation (14), which means that the maximum values assigned to $N_{b}$ retarded the concentration distribution.Figure 18 and Figure 19 aim to report the influence of Casson fluid parameter β and stretching ratio constant γ on entropy generation number $N_{G}$. Figure 18 revealed that an improved total entropy generation distribution is examined when Casson fluid parameters get maximum values. Figure 19 examined that entropy generation rates decrease the function of stretching ratio constant γ.

**Figure 20:**
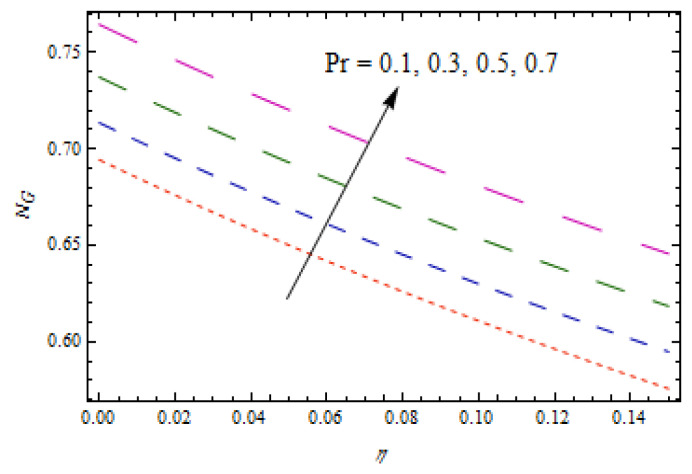
Impact of Pr on $N_{G}.$

**Figure 21:**
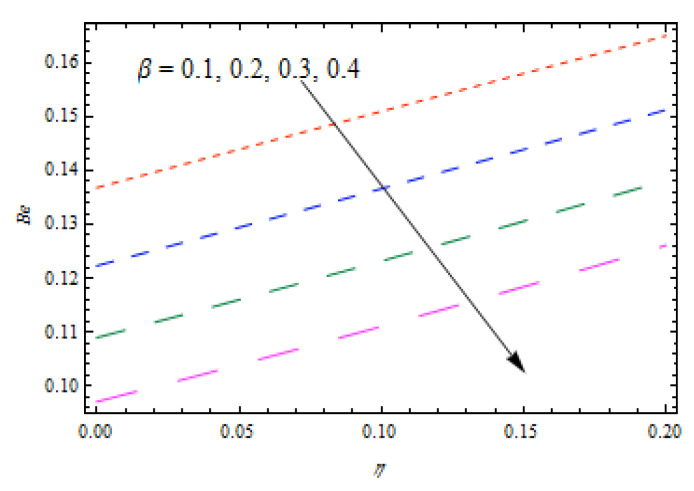
Impact of β on  Be.

**Figure 22:**
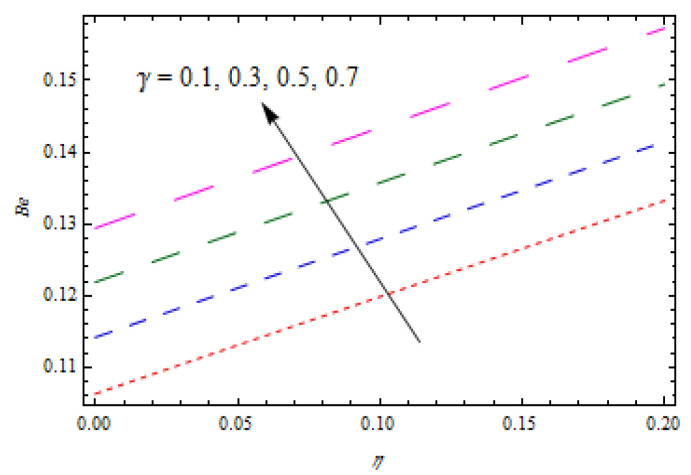
Impact of γ on  Be.

**Figure 23:**
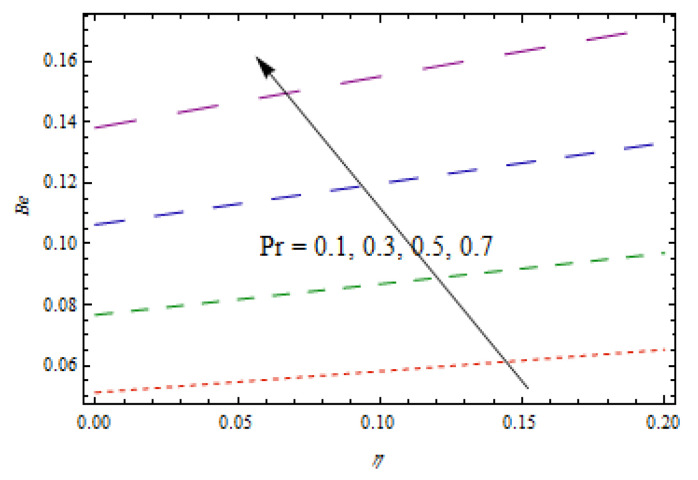
Impact of Pr on  Be.

Figure 20is sketched to observe the impact of Prandtl number Pr  on entropy generation number $N_{G}$. The curve of entropy generation distribution $N_{G}$ attained a maximum level due to maximum values of Pr. The significance of Casson fluid parameter β, stretching ratio γ, and Prandtl number Pr on Bejan number Be are investigated in Figure 21, Figure 22 and Figure 23. Figure 21 shows that Bejan number Be decreases with β. Figure 22 and Figure 23 elucidate that the Bejan number Be increases with stretching ratio constant γ and Pr. The Bejan number is a very important dimensionless number in the entropy generation which reflects the ratio between entropy generations associated with heat transfer to total entropy generation. Usually, the Bejan number reveals numerical values between 0 and 1.In the case that the Bejan number assigns a value close to 1, it means that entropy generation associated with heat transfer is more dominant. To increase the cooling system in the flow of conducting liquids, the Prandtl number can be utilized appropriately.

## 5. Concluding Remarks

- It is noted that the porosity parameter, Casson fluid, and stretching ratio parameter upsurge the radial velocity component.
- The radial component of velocities is increased due to the variation of the porosity parameter and Casson fluid parameter, while the impact of magnetic parameter is reverse.
- Temperature profiles increase for thermophoretic and heat source parameters.
- A declining nanoparticles temperature results from the Prandtl number and heat sink parameter.
- Concentration profile shows increasing behavior for the Lewis number and thermophoretic parameter.
- Increasing values of the stretching ratio and Prandtl number increase the Bejan number, while reverse behavior is observed for the Casson fluid parameter.
- The observations from current analysis can be useful in thermal energy exchange processes, cooling processes, energy consumptions, thermodynamics applications, aircrafts, thermal extrusion systems etc.

## Figures and Tables

**Table 1:** Validation of results with Mustafa [39] when β→∞.

Parameter	$f^{\prime \prime }\left(0\right)$	
M	Mustafa [39]	Present results
0.0	0.259534	0.259538
0.5	0.191176	0.191181

## Nomenclature

$B_{0}$ magnetic field strength	β Casson fluid parameter
µ, dynamic viscosity	p pressure
ρ density	σ electrical conductivity
k permeability of porous medium	T temperature
α thermal diffusivity	$c_{p}$ specific heat
$Q_{0}$ heat generation parameter	$D_{T}$ thermophoretic diffusion coefficient
$D_{B}$ Brownian diffusion coefficient	τ ratio of heat capacity
$k_{0}$ reaction constant	$T_{m}$ mean temperature
Nb Brownian motion parameter	$k^{*}$ coefficient of Rosseland mean absorption
$\sigma^{*}$ constant of Stefan-Boltzmann	γ is stretching ratio
λ heat source parameter	Rd thermal radiation parameter
Re Reynolds number	Pr Prandtl number
Le Lewis number.	M parameter of magnetic
Nb Brownian motion parameter	$D_{B}$ Brownian diffusion parameter
Nt thermophoretic parameter	$\left(\lambda_{1},\lambda_{2}\right)$ slip lengths
S porosity parameter	$D_{T}$ thermophoresis diffusion coefficient
K are thermal conductivity,	$T_{0}$ reference temperature
Φ viscous dissipation	
